# Psychometric performance of the Primary Mitochondrial Myopathy Symptom Assessment (PMMSA) in a randomized, double-blind, placebo-controlled crossover study in subjects with mitochondrial disease

**DOI:** 10.1186/s41687-022-00534-y

**Published:** 2022-12-23

**Authors:** Chad Gwaltney, Jonathan Stokes, Anthony Aiudi, Iyar Mazar, Sarah Ollis, Emily Love, Amel Karaa, Carrie R. Houts, R. J. Wirth, Alan L. Shields

**Affiliations:** 1 Gwaltney Consulting Group, 1 Bucks Trail, Westerly, RI USA; 2 Adelphi Values (or employed at Adelphi Values at time of conduct of research), Boston, MA USA; 3 Stealth BioTherapeutics Inc., Newton, MA USA; 4 Massachusetts General Hospital, Boston, MA USA; 5 Vector Psychometric Group LLC, Chapel Hill, NC USA

**Keywords:** Psychometric evaluation, Patient-reported outcomes, Mitochondrial disease, Primary Mitochondrial Myopathy, Primary Mitochondrial Myopathy Symptom Assessment

## Abstract

**Background:**

The Primary Mitochondrial Myopathy Symptom Assessment (PMMSA) is a 10-item patient-reported outcome (PRO) measure designed to assess the severity of mitochondrial disease symptoms. Analyses of data from a clinical trial with PMM patients were conducted to evaluate the psychometric properties of the PMMSA and to provide score interpretation guidelines for the measure.

**Methods:**

The PMMSA was completed as a daily diary for approximately 14 weeks by individuals in a Phase 2 randomized, placebo-controlled crossover trial evaluating the safety, tolerability, and efficacy of subcutaneous injections of elamipretide in patents with mitochondrial disease. In addition to the PMMSA, performance-based assessments, clinician ratings, and other PRO measures were also completed. Descriptive statistics, psychometric analyses, and score interpretation guidelines were evaluated for the PMMSA.

**Results:**

Participants (N = 30) had a mean age of 45.3 years, with the majority of the sample being female (n = 25, 83.3%) and non-Hispanic white (n = 29, 96.6%). The 10 PMMSA items assessing a diverse symptomology were not found to form a single underlying construct. However, four items assessing tiredness and muscle weakness were grouped into a “general fatigue” domain score. The PMMSA Fatigue 4 summary score (4FS) demonstrated stable test–retest scores, internal consistency, correlations with the scores produced by reference measures, and the ability to differentiate between different global health levels. Changes on the PMMSA 4FS were also related to change scores produced by the reference measures. PMMSA severity scores were higher for the symptom rated as “most bothersome” by each subject relative to the remaining nine PMMSA items (most bothersome symptom mean = 2.88 vs. 2.18 for other items). Distribution- and anchor-based evaluations suggested that reduction in weekly scores between 0.79 and 2.14 (scale range: 4–16) may represent a meaningful change on the PMMSA 4FS and reduction in weekly scores between 0.03 and 0.61 may represent a responder for each of the remaining six non-fatigue items, scored independently.

**Conclusions:**

Upon evaluation of its psychometric properties, the PMMSA, specifically the 4FS domain, demonstrated strong reliability and construct-related validity. The PMMSA can be used to evaluate treatment benefit in clinical trials with individuals with PMM.

*Trial registration* ClinicalTrials.gov identifier, NCT02805790; registered June 20, 2016; https://clinicaltrials.gov/ct2/show/NCT02805790.

**Supplementary Information:**

The online version contains supplementary material available at 10.1186/s41687-022-00534-y.

## Background

Primary mitochondrial diseases (PMD) are a group of rare, clinically heterogeneous disorders resulting from over 350 different genetic mutations of the nuclear DNA (nDNA) and/or mitochondrial DNA (mtDNA) [1–3]. Primary mitochondrial myopathy (PMM) refers to PMD with predominant, but not exclusive, involvement of muscles, leading to defects in oxidative phosphorylation across various muscle groups, including skeletal and cardiovascular muscles [4–6]. Among the PMD population, with an estimated incidence of 1 in 4300 to 10,000 [7–9], it is expected that approximately 90–95% of patients may experience PMM, although the exact prevalence of PMM is unknown [10, 11]. PMM is characterized by a variable signs and symptoms experience, including fatigue, muscle weakness, pain, and exercise intolerance [12, 13]. As a result of this vast array of symptoms, patients report having difficulties with independent and safe ambulation, understanding conversation in noisy settings, driving, personal hygiene, and reading. Social, emotional, and economic concerns also plague adult patients with PMM. In addition, the daily management of symptoms for these patients can be overwhelming. Given this substantial negative impact on aspects of quality of life [14], it is important to consider effects on symptoms when testing novel treatments for this population [15].

To date, there have been no successful PMD clinical trials, partly due to a lack of disease-specific patient outcome measures [16]. Several of the outcomes that characterize PMD are best measured via self-report; however, there is limited use of patient-reported outcome (PRO) symptom measures in PMM studies and existing measures may not be well suited to do so [17, 18]. For example, the Newcastle Mitochondrial Disease Adult Scale (NMDAS) [19] is an assessment of physical functioning and disease severity based on both clinical assessment and patient/caregiver interviews. Although clinician and caregiver perspectives are important, patient self-reports may provide a more direct and accurate measure of symptom severity and function limitations. In addition, The Newcastle Mitochondrial Quality of Life measure (NMQ) [20], a PRO questionnaire, addresses health-related QoL, rather than focusing on the details of the signs and symptoms associated with mitochondrial disease, which may be more important in understanding the direct effects of the disease pathophysiology on the patient. Moreover, while both the NMDAS and NMQ were developed and tested in a mitochondrial disease population, neither was developed specifically for use with individuals with the PMM subtype [19, 20]. Given the heterogeneity of mitochondrial disease, subtype-specific assessments may be warranted for adequate measurement of treatment benefit [17]. Further, the NMDAS is intended for use in six- to twelve-month intervals and the NMQ has a four-week recall period. These relatively long intervals are not well suited to capture the effects of new treatments on symptoms, which may appear in a shorter amount of time. Regulatory guidelines recommend the use of shorter recall periods in PRO measures to be utilized in clinical trials [21, 22]. For example, the Food and Drug Administration Guidance on PROs states that “short recall periods or items that ask patients to describe their current or recent state are usually preferable” [23 (p.14)].

Currently, there is a lack of robust, clinically meaningful, validated clinical trial outcome measures to provide for the optimal efficacy evaluation of novel treatments for patients with PMDs, such as PMM [16]. To fill the gap in available PRO measures that can be used to assess the signs and symptoms of PMM, the Primary Mitochondrial Myopathy Symptom Assessment (PMMSA) was developed. The PMMSA is a ten-item daily assessment evaluating symptom severity over the past 24 h and was developed through extensive patient interviews (42 interviews were conducted) to ensure its content validity. Specifically, the symptoms assessed by the PMMSA were demonstrated to be relevant to individuals with PMM and these individuals understood the instructions, items, and response scales of the measure and were able to provide meaningful responses to the items upon administration [24].

The goal of the current study was to examine the quantitative measurement characteristics of the PMMSA. This included an assessment of the dimensionality of the measure and the reliability, construct validity, and sensitivity to change of the PMMSA scores. This testing was accomplished using data from a Phase 2 randomized, double-blind, placebo-controlled crossover study in subjects with mitochondrial disease in order to inform its inclusion and performance in a subsequent Phase 3 trial. Because mitochondrial disease is a rare disease, the trial included fewer patients than would normally be used for testing of measurement characteristics. Our analyses utilized standard psychometric approaches when possible, with the use of additional innovative methods to account for the relatively small sample size.

## Materials and methods

### Study design

**Fig. 1:**
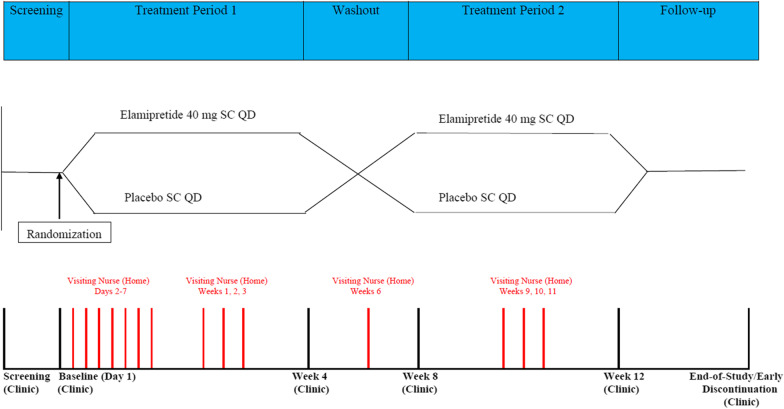
Study schematic. Note: The Primary Mitochondrial Myopathy Symptom Assessment (PMMSA) was administered daily starting at the screening visit. All reference measures (Quality of Life in Neurological Disorders [Neuro-QoL] Fatigue item bank, Physician Global Assessment [PhGA], Patient Global Assessment [PGA], six-minute walk test [6MWT], Triple Timed Up and Go [3TUG] Test, Scale for the Assessment and Rating of Ataxia [SARA]), except for the “most bothersome” item, were administered during each of the study center (“Clinic”) visits. The Neuro-QoL Fatigue item bank was additionally administered during the nurse home visit at Week 6

The PMMSA was administered in a Phase 2 randomized, double-blind, placebo-controlled crossover study to evaluate once daily subcutaneous injections of elamipretide 40 mg in subjects with genetically confirmed mitochondrial disease (ClinicalTrials.gov identifier, NCT02805790; registered June 20, 2016; https://clinicaltrials.gov/ct2/show/‌NCT02805790) (Fig. 1).

### Patient selection criteria

Subjects included in this study provided informed consent prior to participation. To be selected for inclusion, subjects must have met all of the inclusion and none of the exclusion criteria. Broadly, North American male and female consenting adults with a diagnosis of PMM as selected by the Investigators to participate in an earlier Phase 1/2 study of elamipretide were eligible. Those with medical conditions that could put them at risk, who had adverse reactions to the study drug in the Phase 1/2 study, or who were actively enrolled in another trial were excluded [25, 26].

### Assessments

Subjects were asked to complete assessments during study center visits at Screening (Visit 1), Baseline/Day 1 (Visit 2), and at the end of Week 4 (Visit 3), Week 8 (Visit 4), Week 12 (Visit 5), and Week 14 (Visit 6). Additionally, patients completed the PMMSA outside of the clinic via an electronic daily diary for 14 weeks, starting from the Screening Visit and continuing to the End-of-Study (14 weeks) or Early Discontinuation Visit.

#### PMMSA

The PMMSA is a 10-item PRO questionnaire that assesses tiredness at rest, tiredness during activities, muscle weakness at rest, muscle weakness during activities, balance problems, vision problems, abdominal discomfort, muscle pain, numbness, and headache over the previous 24 h on a four-point verbal rating scale (VRS) ranging from 1-Not at all to 4-Severe (Additional file 1: Table S1). As a once-daily diary, subjects completed electronic versions of the PMMSA between 6:00 pm and 11:59 pm beginning with the screening visit and through the end of the study (14 weeks) or until early discontinuation. A principal use of the results presented here was to inform appropriate scoring for the PMMSA.

*Item scores:* Each item’s daily score is reflected by a 1 to 4 VRS where higher scores reflect more severe patient-reported symptom involvement.

*Domain scores:* Two fatigue domains, which resonate with the descriptive language most often used by patients, were hypothesized, including a four-item fatigue scale (4FS) made up of Items 1, (tiredness at rest), 2 (tiredness during activities), 3 (muscle weakness at rest), and 4 (muscle weakness during activities) and a two-item fatigue scale (2FS) made up of Items 2 (tiredness during activities) and 4. (muscle weakness during activities). Both the 4FS and 2FS are derived by summing the item scores for each day. Responses to at least three of the four items in the 4FS and both items in the 2FS were required to calculate a daily score. If the 4FS daily item responses met the noted criteria, prorated summed scores were found by averaging the available item responses and then multiplying that value by the total number of items; the prorated summed score approximates the summed score for the individual if all items had been answered and is equivalent to individual item mean substitution for the missing item responses.

*Total symptom scale (TSS):* The TSS score is calculated as the sum of the 10 items. A prorated summed score was calculated if the patient responded to at least 7 of the 10 items.

*Daily and weekly scores:* Both daily and weekly scores were derived for PMMSA items, domains (4FS and 2FS), and the TSS. Weekly scores were derived as the average daily value from the preceding seven days of a target analysis day. For example, if the Baseline visit (Day 1) is the target analysis day, then the Baseline weekly score is the average of scores generated from study Days 0, − 1, − 2, − 3, − 4, − 5, and − 6.

#### Reference measures

Subjects in the study completed the following assessments which served to support the psychometric evaluation of the PMMSA:

- Quality of Life in Neurological Disorders (Neuro-QoL) Fatigue short form [27]; an eight-item instrument assessing fatigue (five-point VRS ranging from 1-Never to 5-Always); scores were obtained using “look up tables” (i.e., summed score to expected a posteriori score conversion tables) provided by Neuro-QoL;
- Physician Global Assessment (PhGA); a single-item assessment in which the clinician rates the study subject’s overall health status (five-point VRS ranging from 1-Excellent to 5-Poor);
- Patient Global Assessment (PGA), a single-item assessment in which the study subject rates his or her own overall health status (five-point VRS ranging from 1-Excellent to 5-Poor);
- Six-minute walk test (6MWT) [28]; the distance, in meters, that a subject covers during a six-minute period and two self-report items assessing shortness of breath and fatigue before and after the six-minute walk (12-point modified Borg scale);
- Triple Timed Up and Go (3TUG) Test [29]; the time, in seconds, for the subject to complete three repetitions of standing from a seated position, walking 10 feet, and returning to a seated position;
- Scale for the Assessment and Rating of Ataxia (SARA) [30]; an assessment of ataxia in which a clinician rates the subject on eight domains, with higher scores indicating higher ataxia severity;
  - The purpose of including an ataxia severity measure in this study relates to the tendency for this patient population to experience effects on the nervous system which can manifest in balance issues; and
- “Most bothersome item,” a single item assessment prompting subjects to rate the one symptom deemed to be the most bothersome among the 10 symptom concepts assessed in the PMMSA. This item was administered at the Screening Visit only, prior to the first completion of the PMMSA.

All reference measures, except for the “most bothersome” item, were administered during each of the study center visits. The Neuro-QoL Fatigue item bank was additionally administered during the nurse home visit at Week 6. Patients were not compensated specifically for the completion of any of the PRO measures.

### Analyses

All analyses were conducted in SAS 9.4 and focused on evaluating the performance of PMMSA item scores and the a priori PMMSA domain scores. The details are discussed in relevant sections.

#### Descriptive statistics

To examine individual item response distributions and PMMSA summary score properties (4FS, 2FS, and TSS) at the daily level, a subset of collected days (Days − 6 to 0 [the seven days prior to the Baseline visit], Day 1 [Baseline], and each subsequent 10th day through Day 101) were examined. Weekly scores for the PMMSA items, 4FS, 2FS, and TSS (using PMMSA ratings from the seven days preceding a target timepoint [i.e., clinic visits/nurse home visits]) were also calculated.

#### Dimensionality analysis

As a newly developed, potentially multi-dimensional scale, PMMSA data were summarized to see if stable patterns among items emerged in order to increase understanding of the scale structure and inform future scoring of the tool. Due to the relatively small sample size, a repeated measures approach was used that capitalized on multiple data points from the same patient. Specifically, daily responses for Day -6, Day 1, and every subsequent 10^th^ day were combined into a dimensionality analyses dataset. These days were selected to capture pre-intervention days and then, post-intervention, were selected to reduce the potential for auto-correlations between days close together temporally. This data was used to generate polychoric correlations among the PMMSA items and were also submitted to categorical exploratory factor analysis (CEFA), which adapts typical continuous variable factor analytic methods to appropriately accommodate categorical data (such as the PMMSA 4-category response options) [31]. While the sampling of multiple days will produce biased standard errors (due to the non-independence of observations), it provides unbiased point estimates (e.g., factor loadings), allowing for a general review of the likely underlying measurement structure of the PMMSA items.

#### Reliability analysis

The reliability of PMMSA scores was assessed in two ways. First, internal consistency estimates of the PMMSA TSS, 4FS, and 2FS were assessed via Cronbach’s coefficient alpha (α) generated at each of the daily analysis time points (Day − 6, Day 1, and every subsequent 10th day up to Day 101]).

Second, test–retest reliability estimates for PMMSA item, TSS, 4FS, and 2FS scores were calculated as the Pearson correlation coefficients relating weekly scores generated during (1) screening Week 1 (Days − 13 to − 7) and screening Week 2 (Days − 6 to 0) and (2) Week 7 and Week 8. These times were selected because patients were expected to have stable symptoms during these intervals. Internal consistency and test–retest reliability were examined. Reliability estimates at or greater 0.70 was considered acceptable [35].

#### Construct-related validity analysis

First, convergent and discriminant validity was assessed by cross-sectional Pearson’s and Spearman’s correlation coefficients generated between weekly scores produced by the PMMSA and the reference measures administered at Visits 2 through 6. Second, known-groups analyses were planned by comparing the PMMSA weekly scores within pre-specified groupings determined by the PhGA, PGA, and 6MWT scores via independent samples t-tests within each time point. Specifically, known groups were defined by (1) PGA scores (two groupings comprising those who self-report their health status as “Excellent” or “Very Good” and “Poor” or “Fair”), (2) PhGA scores (two groupings comprising subjects with clinician-rated health status as “Excellent” or “Very Good” and “Poor” or “Fair”), and (3) 6MWT results (two groupings comprising subjects who covered 400 m or more and those who covered less than 400 m) [28]. It was expected that the Excellent/Very Good groups (for the PGA and PhGA analysis) and the ≥ 400 m group (for the 6MWT analysis) would have lower PMMSA weekly scores. Third, sensitivity-to-change estimates were generated to reflect the relationships between weekly PMMSA score changes and change scores observed in the relevant reference measures via Pearson’s and Spearman’s correlation. The change scores for all relevant measures were generated from the end of the experimental treatment period to the end of the placebo period two.

#### Score interpretation analysis

Score interpretation analysis informs the clinical meaning that may be attached to observed within-person change. Distribution-based methods and anchor-based methods were used to inform the interpretation of scores and arrive at treatment responder definitions [32]. Distribution-based methods included the 0.5 standard deviation (SD) and standard error of measurement (SEM) [33]. The anchor-based methods used the change in PGA, PhGA, and 6MWT scores as anchors to categorize patients into improved and non-improved groups; the mean changes in PMMSA scores in the improved groups on the anchor measures were reported as candidate responder definitions [34]. The distribution-based methods are presented to provide some information on the degree of variability in the measures at baseline. The responder definitions for the PMMSA scores would be expected to exceed the values of the 0.5 SD and SEM.

## Results

*Sample demographics:* Table 1 presents patient demographics and genotypic characteristics. Thirty-one individuals participated in the SPIMM 202 clinical trial, 30 of whom completed all study activities. The 30 participants had a mean age of 45.30 years, with 83.0% (n = 25) being female and 97.0% being non-Hispanic white.

**Table 1:** Demographic and genotypic characteristic summary (N = 30) [25]

Variable	n	Mean (SD or range) or percentage
Age (in years)	30	45.3 (17–65)
Sex		
Female	25	83%
Male	5	17%
Race (one participant chose all that applied)		
White	30	100%
Asian	1	3%
Ethnicity		
Not Hispanic of Latino	29	97%
Hispanic of Latino	1	3%
Weight (kg)	30	65.1 (± 14.2)
BMI (kg/m^2^)	30	24.1 (15.8–36.0)
Baseline 6MWT (m)	30	389.4 (± 23.6)
Baseline 6MWT (m) < 450	22	73%
Baseline 6MWT (m) ≥ 450	8	27%
Genotypic characteristics		
* Mitochondrial DNA (mtDNA)*		
Disorders involving mtDNA mutations that impair mitochondrial protein synthesis *in toto*	19	63%
Mitochondrial deletion syndrome	11	37%
m.3243A > G	4	13%
m.8344A > G	3	10%
Multisystem mitochondrial disorder (MT-TH and tRNA)	1	3%
Disorders involving mtDNA mutations that affect the subunits of the respiratory chain	5	17%
Multisystem mitochondrial disorder (MT-COX1)	1	3%
Mitochondrial Myopathy (MTCYB)	1	3%
LHON Plus	1	3%
Multisystem Mitochondrial Disorder (MT-ND3)	1	3%
Leigh syndrome (NDUFV1)	1	3%
*Nuclear DNA (nDNA)*		
Disorders involving nDNA mutations causing defects of intergenomic signaling	3	10%
POLG-related disorder	3	10%
Disorders involving nDNA mutations causing alterations of the lipid milieu of the inner mitochondrial membrane	1	3%
MEGDEL	1	3%
Disorders involving nDNA mutations causing alterations of mitochondrial motility or fission	2	7%
Multisystem mitochondrial disorder (OPA1)	2	7%

One subject failed to meet inclusion criteria at Visit 2 and was discontinued from the study. One subject discontinued from the study after the Week 10 home visit (between Visit 4 and Visit 5). Both participants’ demographic information collected at screening is included in the above summaries

6MWT = six-minute walk test; SD = standard deviation

*PMMSA item, domain (4FS and 2FS), and TSS scores:* Descriptive statistics for the 10 PMMSA item scores were calculated for Days -6 to 0, Day 1, and each subsequent 10th day through Day 101. The distributions of daily response showed that the patients used the full range of the 1–4 response scale. Items assessing vision problems, abdominal pain, numbness, and headache were generally rated as lower in severity (response means ranged from 1.55 [SD = 0.81] to 2.12 [SD = 1.04] across all analyzed days), while items assessing tiredness at rest, tiredness during activities, muscle weakness at rest, muscle weakness during activities, balance problems, and muscle pain were generally rated as “Mild” or “Moderate” in severity (response means ranging from 2.36 [SD = 0.98] to 2.86 [SD = 0.81] considering all analyzed days). Similarly, and consistent with expectations that not all PMD patients will experience all PMD symptoms, all items were endorsed as “not at all” by at least one subject on each day prior to Baseline. At Baseline, subjects endorsed “not at all” for the items assessing vision problems (n = 13, 43.3%), abdominal discomfort (n = 13, 43.3%), numbness (n = 17, 56.7%), and headache (n = 19, 63.3%).

The weekly PMMSA item, domain (4FS and 2FS), and TSS scores are presented in Table 2. In general, weekly score averages for items assessing tiredness, muscle weakness, balance problems, vision problems, and muscle pain were between moderate and severe, whereas weekly averages for items assessing abdominal discomfort, numbness, and headache were between mild and moderate. Overall, the 4FS and 2FS severity scores were slightly higher than the TSS, when considering the number of items contributing to each score; however, all weekly summary scores were generally between “mild” to “moderate” on the response scale.

**Table 2:** PMMSA weekly scores (mean [SD])

Item	Baseline	Week 4 Treatment 1 end	Week 6 Nurse home visit	Week 8 Washout end	Week 12 Treatment 2 end	Week 14 Two weeks post-treatment 2
	(N = 30)	(N = 29)	(N = 29)	(N = 30)	(N = 28)	(N = 28)
Tiredness at rest	2.82 (0.66)	2.55 (0.70)	2.69 (0.62)	2.73 (0.65)	2.45 (0.74)	2.63 (0.72)
Tiredness during activities	3.06 (0.63)	2.61 (0.70)	2.94 (0.63)	2.89 (0.73)	2.63 (0.78)	2.77 (0.70)
Muscle weakness at rest	2.49 (0.71)	2.29 (0.77)	2.51 (0.75)	2.54 (0.69)	2.28 (0.68)	2.49 (0.74)
Muscle weakness during activities	2.93 (0.71)	2.54 (0.74)	2.86 (0.73)	2.87 (0.75)	2.63 (0.77)	2.79 (0.65)
Balance problems	2.53 (0.78)	2.44 (0.82)	2.51 (0.80)	2.46 (0.77)	2.38 (0.77)	2.56 (0.76)
Vision problems	2.04 (1.01)	2.06 (0.97)	2.22 (0.99)	2.29 (1.00)	2.17 (0.95)	2.26 (1.02)
Abdominal discomfort	1.82 (0.79)	1.82 (0.67)	1.94 (0.70)	1.90 (0.78)	1.85 (0.78)	1.94 (0.89)
Muscle pain	2.33 (0.94)	2.20 (0.86)	2.34 (0.85)	2.40 (0.94)	2.27 (0.88)	2.47 (0.89)
Numbness	1.70 (0.86)	1.79 (0.92)	1.67 (0.82)	1.84 (0.93)	1.73 (0.74)	1.91 (0.86)
Headache	1.61 (0.70)	1.43 (0.52)	1.67 (0.67)	1.65 (0.71)	1.49 (0.54)	1.71 (0.74)
TSS	23.33 (5.12)	21.74 (5.08)	23.35 (4.63)	23.56 (5.23)	21.88 (5.02)	23.53 (5.29)
4FS	11.30 (2.50)	10.00 (2.77)	11.00 (2.55)	11.02 (2.63)	9.99 (2.84)	10.68 (2.68)
2FS	5.99 (1.30)	5.15 (1.41)	5.80 (1.31)	5.76 (1.45)	5.26 (1.52)	5.56 (1.32)

PMMSA 10-item weekly scores have a possible range of 10 to 40. PMMSA Fatigue 4 weekly scores have a possible range of 4 to 16. PMMSA 2-item weekly scores have a possible range of 2 to 8. Individual item weekly averages may range from 1 to 4

2FS = two-item fatigue score; 4FS = Fatigue 4 Scale; PMMSA = Primary Mitochondrial Myopathy Symptom Assessment; SD = standard deviation; TSS = total symptom scale

*Inter-item correlations:* As can be seen in Table 3, there is a strong item cluster among the first 4 PMMSA items (tiredness at rest, tiredness during activities, muscle weakness at rest, muscle weakness during activities) in which those items are more interrelated to each other than to the other items in the assessment; this is not unexpected given the similarity/repeated nature of item text/content and supports the a priori 4FS score. PMMSA item 5 (balance problems) is also related to this cluster, but at a lower level.

**Table 3:** Average polychoric correlations among PMMSA items

Item Content	Tiredness at rest	Tiredness during activities	Muscle weakness at rest	Muscle weakness during activities	Balance problems	Vision problems	Abdominal discomfort	Muscle pain	Numbness	Headache
Tiredness at rest	1.00									
Tiredness during activities	0.83	1.00								
Muscle weakness at rest	0.82	0.74	1.00							
Muscle weakness during activities	0.74	0.81	0.85	1.00						
Balance problems	0.48	0.53	0.56	0.59	1.00					
Vision problems	0.08	0.13	0.13	0.19	0.32	1.00				
Abdominal discomfort	0.37	0.31	0.29	0.28	0.23	0.26	1.00			
Muscle pain	0.38	0.41	0.45	0.51	0.24	0.07	0.49	1.00		
Numbness	0.29	0.34	0.34	0.43	0.46	0.29	0.28	0.59	1.00	
Headache	0.31	0.20	0.21	0.26	0.04	0.22	0.52	0.41	0.07	1.00

Presented as average polychoric correlation coefficients among PMMSA item scores across all assessment days (i.e., the analysis ignores time of assessment and averages scores generated from daily responses for Day -6 [from screening], Day 1 [Baseline], and every subsequent 10th day [Days 11, 21, and up to Day 101])

PMMSA = Primary Mitochondrial Myopathy Symptom Assessment

*Categorical exploratory factor analysis*: Table 4 presents results of the exploratory factor analyses of the PMMSA items for both the full 10-item set and the a priori defined 4FS item set (factor loadings > 0.40 have been bolded for ease of review) [35]. For the 10-item set, the two-factor solution suggests item clusters of two factors comprising Items 1–5 and 7–10, respectively. However, the domain created by items 7–10 was not considered conceptually coherent and interpretable and was not examined further. For the a priori 4FS item set, all items loaded strongly onto a single factor but there were also strong loadings for the 2 tiredness items on the second factor in the 2-factor solution, indicating that there may be residual dependence among these 2 items (and likely the muscle weakness items) due to content similarity. However, this finding does not preclude these items creating meaningful and useful summed scores within the classical test theory framework.

**Table 4:** Item loadings and factor correlations from exploratory factor analyses of the PMMSA items, for both the 10-item TSS and 4FS

Item	F1	F1	F2		F1	F2	F3	
TSS								
Tiredness at rest	**0.88**	**0.93**	− 0.07		− 0.15	**− 0.93**	0.12	
Tiredness during activities	**0.89**	**0.92**	− 0.03		− 0.03	**− 0.92**	− 0.02	
Muscle weakness at rest	**0.92**	**0.92**	0.01		0.01	**− 0.92**	− 0.02	
Muscle weakness during activities	**0.94**	**0.86**	0.13		0.16	**− 0.86**	− 0.02	
Balance problems	**0.62**	**0.62**	0.01		0.30	**− 0.54**	− 0.18	
Vision problems	0.16	0.04	0.18		0.29	0.03	0.10	
Abdominal discomfort	0.37	0.02	**0.58**		0.16	− 0.05	**0.66**	
Muscle pain	**0.57**	− 0.01	**0.89**		**0.49**	− 0.13	**0.42**	
Numbness	**0.45**	0.07	**0.60**		**0.87**	− 0.01	0.02	
Headache	0.28	− 0.02	**0.49**		− 0.06	0.00	**0.80**	
		Factor correlations			Factor correlations			
			F1	F2		F1	F2	F3
		F1	1		F1	1		
		F2	0.55	1	F2	− 0.43	1	
					F3	0.23	0.32	1

Item	F1	F1	F2	
FS				
Tiredness at rest	**0.90**	0.01	**− 1.01**	
Tiredness during activities	**0.90**	**0.47**	**− 0.49**	
Muscle weakness at rest	**0.92**	**0.57**	− 0.40	
Muscle weakness during activities	**0.92**	**1.03**	0.04	
		Factor correlations		
			F1	F2
		F1	1	
		F2	− 0.78	1

Bold text indicates factor loadings larger than 0.40 in absolute value

CEFA = categorical exploratory factor analysis; PMMSA = Primary Mitochondrial Myopathy Symptom Assessment

The CEFA is based on scores combined across all assessment days (i.e., the analysis ignores time of assessment and averages scores generated from daily responses for Day − 6 [from screening], Day 1 [Baseline], and every subsequent 10th day [Days 11, 21, and up to Day 101]). Nesting was intentionally ignored. Additionally, in instances in which a response category had less than five observed responses, the responses were recoded as the next lowest response category except for the response option 0-Not at all, which was recoded as the next highest response category. For the CEFA of the 10-item PMMSA, solutions with up to three factors were examined, while a maximum of two factors were used for the Fatigue 4 scale subset. An item was considered to belong to a factor if the loading value was greater than 0.40 in absolute magnitude

Based on the results of the inter-item correlations and the factor analysis, the PMSSA TSS and was dropped from further consideration. Additionally, the 2FS can be considered a less accurate version of the 4FS and preference was given to the 4FS which contained more items and therefore more information.

### Reliability analyses

*Internal consistency:* As described in them Methods, coefficient alpha for the 4FS scores were computed across the 12 individual days selected for the day-level analyses. The mean alpha across these days was 0.90 (median = 0.91, SD = 0.04, range: 0.82–0.94)), demonstrating stability of the alpha estimate despite the limited sample size and providing evidence that the internal consistency reliability of the 4FS scores is at an acceptable level.

*Test–retest reliability:* Table 5 presents a stable test–retest reliability of the weekly 4FS scores for the two periods described in the Methods section. The reliability values were above the threshold considered sufficient to support their use in making individual-level decisions (0.90) and group-level comparisons (0.80). The sample size for Period 1 was very small because not all patients completed a sufficient number of daily assessments during the screening period Week 1 to generate weekly scores.

**Table 5:** Test–retest reliability of the PMMSA Scores

	Period 1^*^ (N = 12)	Period 2^†^ (N = 28)
4FS	0.96	0.91
Tiredness at rest	0.88	0.81
Tiredness during activities	0.94	0.85
Muscle weakness at rest	0.90	0.90
Muscle weakness during activities	0.98	0.92
Balance problems	0.82	0.92
Vision problems	0.98	0.96
Abdominal discomfort	0.93	0.93
Muscle pain	0.94	0.95
Numbness	0.96	0.98
Headache	0.80	0.81

4FS = 4-item Fatigue Scale; PMMSA = Primary Mitochondrial Myopathy Symptom Assessment; TRT = test–retest

^*^Test–retest estimates were calculated as the Pearson correlation coefficients relating weekly scores generated during screening Week 1 (Days − 13 to − 7) and screening Week 2 (Days − 6 to 0)

^†^Test–retest estimates were calculated as the Pearson correlation coefficients relating weekly scores generated during Study Week 7 and Week 8 (last two weeks of the washout period)

### Construct-related validity analyses

Table 6 presents the average correlations between the weekly 4FS, the individual PMMSA items, and scores produced by the administered reference measures; correlations were assessed cross-sectionally by visit but in the interest of space, we report the mean correlation coefficient across the examined visits. We note that the correlations intended to establish convergent validity were consistent with respect to direction and generally consistent with respect to magnitude across visits (e.g., between 4FS and fatigue item from the 6WMT task observed r’s = 0.29, 0.47, 0.35, 0.32, 0.23 across 5 examined visits). The mean correlations, excepting those involving the Neuro-QoL fatigue scale, are based on the average of coefficients generated at Study Visits 2 (N = 30), 3 (N = 29), 4 (N = 30), 5 (N = 28), and 6 (N = 28). For correlations involving the Neuro-QoL fatigue scale, mean correlations included an additional nurse home visit at Week 6 (N = 27). As indicators of discriminant validity, correlations among the 4FS and height, weight, and BMI tended to be near-zero; the most discrepant results with respect to this was the correlation of PMMSA item 8 (muscle pain) with weight and BMI (both mean r = 0.41).

**Table 6:** Average correlations between reference variables and PMMSA scores across time points

	4FS	Item 1 Tiredness at rest	Item 2 Tiredness during activities	Item 3 Muscle weakness at rest	Item 4 Muscle weakness during activities	Item 5 Balance problems	Item 6 Vision problems	Item 7 Abdominal discomfort	Item 8 Muscle pain	Item 9 Numbness	Item 10 Headache
Height	− 0.15	− 0.28	− 0.03	− 0.18	− 0.01	0.07	0.02	− 0.05	− 0.06	0.18	− 0.17
Weight	− 0.01	− 0.04	0.11	− 0.07	0.02	− 0.04	− 0.03	0.00	0.41	0.39	0.00
BMI	0.05	0.06	0.08	0.01	0.03	− 0.06	0.01	0.02	0.41	0.29	0.09
*Reference measures*											
Neuro-QoL Fatigue	0.69	0.63	0.67	0.62	0.67	0.35	0.01	0.36	0.31	0.14	0.25
PhGA	0.28	0.36	0.32	0.38	0.41	0.34	0.16	0.36	0.25	0.16	0.26
PGA	0.39	0.27	0.27	0.29	0.33	0.30	0.02	0.03	0.11	0.20	− 0.03
6MWT PRO fatigue (pre)	0.43	0.43	0.39	0.44	0.43	0.15	− 0.15	0.30	0.43	0.22	0.14
6MWT PRO dyspnea (pre)	0.26	0.30	0.19	0.32	0.18	0.21	0.02	0.04	0.11	0.06	− 0.13
6MWT distance	− 0.13	− 0.06	− 0.08	− 0.14	− 0.19	− 0.32	− 0.17	0.16	0.00	− 0.18	0.19
6MWT PRO fatigue (post)	0.33	0.35	0.30	0.32	0.33	0.09	0.02	0.05	0.18	− 0.05	0.08
6MWT PRO dyspnea (post)	0.24	0.27	0.18	0.24	0.22	0.26	0.12	− 0.12	− 0.04	− 0.16	− 0.02
3TUG time to completion	0.11	0.01	0.08	0.08	0.21	0.39	0.39	− 0.03	0.11	0.39	− 0.11
SARA	0.25	0.22	0.18	0.30	0.27	0.50	0.15	− 0.04	− 0.12	0.22	− 0.14

All correlations except those involving the Neuro-QoL fatigue scale are based on the average of coefficients generated across five time points including Study Visits 2 (N = 30), 3 (N = 29), 4 (N = 30), 5 (N = 28) and 6 (N = 28). For correlations involving the Neuro-QoL fatigue scale, scores were generated from an additional nurse home visit at Week 6 (N = 27). In each instance, PMMSA scores are based on weekly averages

3TUG = Triple Timed Up and Go test; 6MWT = six-minute walk test; Neuro-QoL = Quality of Life in Neurological Disorders; PGA = Patient Global Assessment; PhGA = Physician Global Assessment; PMMSA = Primary Mitochondrial Myopathy Symptom Assessment; SARA = Scale for the Assessment and Rating of Ataxia. PhGA, PGA, and 6MWT PRO Borg items are assessed using Spearman’s correlations; all other reported correlations are Pearson’s

With respect to convergent validity, several a priori hypotheses were confirmed [36, 37].

- A positive and strong correlation (r = 0.69) was found between the 4FS and the Neuro-QoL indicators of fatigue.
- Consistently positive and mostly moderate correlations (r = 0.30 to 0.49) were found between 4FS and the 6MWT self-reported fatigue score both pre- and post-6MWT.
- Correlation between 4FS and self-reported overall health status (as assessed by the PGA, r = 0.39) was stronger than the physician ratings of the subject’s health status (as assessed by the PhGA, r = 0.28).
- A positive and small correlation (r = 0.25) was observed between the 4FS and scores from SARA. Although not a primary consideration for this analysis, a strong positive correlation was found between the SARA and the PMMSA balance item, as anticipated.
- Small correlations were observed between the 4FS and 3TUG time to completion and 6MWT distance traveled score. These correlations were also in the expected directions (i.e., positive for the 3TUG completion time and negative for the 6MWT distance).

Overall, the PMMSA 4FS scores tended to correlate more strongly with fatigue-specific reference variables and were found to have less strong correlations with more distal reference variables (e.g., PhGA, 3TUG time to completion).

The most bothersome symptom item from among the PMMSA items was also evaluated. A total of seven unique symptoms were reported as most bothersome, including muscle weakness during activities (n = 7 or 23.3%), balance problems (n = 6 or 20%), vision problems (n = 5 or 16.7%), tiredness during activities (n = 5 or 16.7%), tiredness at rest (n = 3 or 10%), abdominal discomfort (n = 2 or 6.7%), and muscle pain (n = 2 or 6.7%). No subjects reported muscle weakness at rest, numbness, or headache as most bothersome. The reported severity of the most bothersome symptom at Baseline on the PMMSA (mean = 3.10, SD = 0.80) was greater than the severity of all other individual symptoms combined (mean = 2.35, SD = 0.60). The latter result was replicated when considering all PMMSA response days, in which the mean response for the most bothersome symptom was 2.88 (SD = 0.82) compared to 2.18 (SD = 0.56) for the remaining nine items.

*Known-groups analyses:* Table 7 presents the average scores on each PMMSA variable across the observation weeks. Due to the small sample sizes in each group standardized differences were reviewed but no inferential tests were performed. As expected, more positive global ratings were associated with numerically lower weekly PMMSA scores and the magnitudes of the relationships were generally strong, particularly for the PGA. The relationship with the 6MWT grouping was not as strong and in the unexpected direction for two PMMSA items (abdominal discomfort and headache).

**Table 7:** Known-groups comparisons

	4FS	Item 1 Tiredness at rest	Item 2 Tiredness during activities	Item 3 Muscle weakness at rest	Item 4 Muscle weakness during activities	Item 5 Balance problems	Item 6 Vision problems	Item 7 Abdominal discomfort	Item 8 Muscle pain	Item 9 Numbness	Item 10 Headache
PhGA scores: Fair/Poor versus Excellent/Very Good											
Poor PhGA (n = 7–14)*	11.2	2.8	2.9	2.6	3.0	2.7	2.1	2.0	2.4	2.0	1.6
Good PhGA (n = 2–6)*	8.0	2.0	2.1	1.8	2.1	1.8	2.0	1.8	2.1	1.4	1.8
Difference†	3.2	0.8	0.8	0.8	0.9	0.9	0.1	0.2	0.4	0.6	-0.2
PGA scores: Fair/Poor versus Excellent/Very Good											
Poor PGA (n = 14–20)*	11.4	2.8	3.0	2.59	3.0	2.7	2.3	2.0	2.5	1.9	1.7
Good PGA (n = 1–5)*	8.1	2.1	2.2	1.76	2.1	1.8	1.9	1.3	1.6	1.5	1.2
Difference^†^	3.2	0.7	0.8	0.83	0.9	0.8	0.4	0.7	0.9	0.4	0.5
6MWT: < 400 m versus ≥ 400 m											
< 400 m 6MWT group (n = 12–17)*	10.9	2.7	2.8	2.5	2.9	2.6	2.3	1.8	2.3	1.9	1.5
≥ 400 m 6MWT group (n = 12–17)*	10.4	2.6	2.8	2.3	2.7	2.3	2.1	2.0	2.3	1.7	1.7
Difference^†^	0.5	0.0	0.1	0.2	0.2	0.3	0.2	-0.1	0.0	0.3	-0.1

4FS = Fatigue 4 Scale; 6MWT = six-minute walk test; PGA = Patient Global Assessment; PhGA = Physician Global Assessment; SD = standard deviation

*Average

^†^Mean group difference averaged over time at examined visits

*Sensitivity-to-change analysis:* Table 8 presents the correlations among change scores between weekly PMMSA 4FS scores and reference variables, with change defined as the difference in scores between the active treatment period (Visit 3 or 5, depending on order) and the placebo treatment period (Visit 3 or 5, depending on order). Indicators of score sensitivity include consistently (1) positive and strong correlation (r = 0.71) between change in the 4FS and change in Neuro-QoL fatigue scores, (2) negative and moderate correlation (r = − 0.46) between change in the 4FS and change in 6MWT distance, and (3) positive and small correlation (r = 0.21) between change in the 4FS and change in the 3TUG time-to-completion results. The small correlations between change in the 4FS and change in PhGA and PGA may be due to a lack of variability in the PhGA ratings over time. The correlations of change in PMMSA item scores were generally in the expected direction; however, correlations between the PMMSA items were typically not as strong as those observed with the 4FS.

**Table 8:** Correlations between weekly PMMSA change scores and reference variable change scores (N = 27)

	4FS	Item 1 Tiredness at rest	Item 2 Tiredness during activities	Item 3 Muscle weakness at rest	Item 4 Muscle weakness during activities	Item 5 Balance problems	Item 6 Vision problems	Item 7 Abdominal discomfort	Item 8 Muscle pain	Item 9 Numbness	Item 10 Headache
Neuro-QoL Fatigue	0.71^*^	0.72	0.70	0.58	0.62	0.64	0.46	0.21	0.47	0.23	0.35
PhGA	− 0.04	− 0.03	− 0.05	− 0.08	0.06	− 0.24	0.16	− 0.22	0.08	− 0.3	0.22
PGA	0.13	0.23	0.14	0.14	0.21	0.03	0.15	0.29	0.15	− 0.22	0.04
6MWT distance	− 0.46^†^	− 0.37	− 0.44	− 0.53	− 0.35	− 0.31	− 0.26	− 0.01	− 0.33	− 0.08	− 0.28
3TUG time to completion	0.21	0.24	0.17	0.28	0.07	0.18	− 0.03	− 0.06	0.11	0.16	0.01

^*^p < 0.001 and ^†^*p* < 0.05

3TUG = Triple Timed Up and Go test; 4FS = Fatigue 4 Scale; 6MWT = six-minute walk test; Neuro-QoL = Quality of Life in Neurological Disorders; PGA = Patient Global Assessment; PhGA = Physician Global Assessment; PMMSA = Primary Mitochondrial Myopathy Symptom Assessment. PhGA, PGA, and 6MWT PRO Borg items are assessed using Spearman’s correlations; all other reported correlations are Pearson’s

### Score interpretation guidelines

Table 9 presents results from the distribution- and anchor-based analysis. The meaningful change threshold estimates for the 4FS ranged from 0.79 (1 SEM) to 2.14 (PGA anchor), with a median value of 2.05. The results generally suggest that change of approximately 2 points on the 13-point 4FS (range of 4–16) could be considered relevant to patients. For the individual non-fatigue items, the median estimates were all below 1 unit on the four-point response scales, with a range of 0.06 (numbness) to 0.38 (muscle pain).

**Table 9:** Candidate clinically important differences for 4FS and individual PMMSA items

Measure	Distribution-based methods		Anchor-based methods			
	½ SD	SEM	PGA	PhGA	6MWT	Median
4FS weekly score	1.25	0.79	2.14	1.53	2.05	2.05
Tiredness at rest	0.33	0.29	0.46	0.31	0.55	0.46
Tiredness during activities	0.32	0.25	0.56	0.4	0.62	0.56
Muscle weakness at rest	0.36	0.22	0.47	0.36	0.39	0.39
Muscle weakness during activities	0.36	0.2	0.65	0.47	0.49	0.49
Balance problems	0.39	0.22	0.27	0.03	0.35	0.27
Vision problems	0.51	0.2	0.23	0.04	0.24	0.23
Abdominal discomfort	0.29	0.21	0.29	0.07	0.26	0.26
Muscle pain	0.47	0.21	0.55	0.22	0.38	0.38
Numbness	0.43	0.31	–	0.04	0.07	0.06
Headache	0.43	0.18	0.34	0.07	0.61	0.34

–indicates a logically inconsistent value was found, in which improved patients had worse PMMSA item scores, and this value was excluded as a candidate threshold value

4FS = Fatigue 4 Scale; 6MWT = six-minute walk test; PGA = Patient Global Assessment; PhGA = Physician Global Assessment; SD = standard deviation; SEM = standard error of measurement

## Discussion

The PMMSA is a PRO daily diary measure that was created to evaluate treatment benefit in regulated PMM clinical trials and developed in accordance with best measurement practices and regulatory guidelines [21–23]. With its content validity established [24], results from the present analyses were generated to evaluate the measure’s underlying factor structure, scoring algorithm and address its psychometric performance. The CEFA results suggest that the full 10-item PMMSA is multidimensional and a composite or TSS may not be appropriate for this instrument. However, scale dimensionality analyses did suggest the first four items of the PMMSA form a general fatigue item parcel. As the remaining six items were not included as an a priori domain, did not load strongly on a single factor, it is more appropriate to treat them as individual item scores. Therefore, the PMMSA is best represented by 6 scores: fatigue (four-item composite), balance problems, vision problems, abdominal discomfort, muscle pain, numbness, and headache.

Results support the conclusion that the PMMSA yields scores that are reliable, valid, and sensitive to change over time. Test–retest reliability remained stable producing similar result between the two different assessment points. The pattern of correlations with other variables indicated convergent and discriminant validity: For example, while the PMMSA weekly fatigue score was robustly related to the NeuroQoL Fatigue measure, it was largely unrelated to unrelated concepts, such as height, weight, and ataxia. Specific correlations between select individual items and criterion measures—i.e. the balance problems item and the SARA—also supported the validity of the measure. However, the headache item was not correlated with any of the criterion measures. Known-groups analyses indicated that the PMMSA weekly fatigue scores were robustly related with patient and physician global evaluations of the patient’s health. Individual items were also generally related to the global evaluations as expected, particularly with the patient’s global assessment. The change in the weekly PMMSA fatigue scores was also strongly associated with change over time on the NeuroQoL Fatigue measure and 6MWT distance. Small to moderate correlations were observed between the other PMMSA weekly scores and the criterion variables. Overall, strong evidence for the measurement characteristics of the PMMSA scores was obtained from the trial, despite the small sample size.

The responder definition analysis indicated that a change of approximately 2 points on the PMMSA weekly fatigue score could be considered meaningful for patients. The average baseline score for the 4FS was 11.3. Therefore, a 2-point improvement represents a 15–20% reduction in the fatigue score. Similarly, the meaningful change thresholds for the individual item scores were generally between 0.25 and 0.50, which reflect 15–20% reductions from baseline. The estimates for the 4FS and tiredness and muscle weakness items exceeded the distribution-based values; this is expected as the distribution-based approaches are group-level analyses and likely underestimate the true responder threshold. However, this was not the case for the other individual items, where the 0.5 SD values were larger than the responder definition estimate. Therefore, these specific item-level estimates should be confirmed in future studies with larger sample sizes and tailored anchor measures, if possible. The responder definition estimates are based on anchor-based analyses that follow regulatory recommendations [23]. However, different methods that are designed to identify meaningful within-patient change (e.g., [38]), could yield different results. Change from baseline on the 6MWT was the only anchor variable that was associated with PMMSA scores above 0.30, a common threshold for identifying suitable anchors [39]; this may be due to limited variability in the patient and clinician global measures. These relatively low correlations may reduce the precision and reliability of the meaningful change estimates.

The heterogeneity of the PMM symptom experience and the challenges in detecting treatment effects using a multi-symptom assessment in this context are formidable. In this context, supplementing the PMMSA with an item that asked subjects to select which PMM symptom they deemed most bothersome at screening was an important aspect of the overall measurement strategy. A total of seven unique symptoms were reported as most bothersome, with muscle weakness during activities being reported most often. Importantly, symptom severity was rated as higher for the endorsed most bothersome symptom relative to other symptoms. This additional item serves to tailor the PMMSA to the individual’s experience and could be explored further to determine whether an individual-specific single item could be used in conjunction with a more general mitochondrial disease assessment for future research.

The results presented herein ought to be interpreted with caution and in the context of several limitations. First, many of the analyses were conducted using samples too small to test many of the underlying methodological assumptions and, therefore, not all types of analyses could be implemented and the magnitude of the relationships between variables were reviewed for consistency with a priori hypotheses but rarely tested for statistical significance. These challenges are common when developing PRO measures for rare disease populations [40]. Nevertheless, the analyses produced plausible patterns of results, even suggesting discriminant patterns of relationships between the PMMSA and the criterion variables. In addition, the sample was limited to United States-based, English-speaking participants, lacking cultural diversity in representation of mitochondrial disease populations. To address these limitations, it is recommended that future research assesses the PMMSA among individuals globally [41]. Future studies could also consider the value of other methods for summarizing the daily data, such as examining the most severe score in a week, rather than the average score. Additionally, the PMMSA and other PRO measures were completed in the context of a carefully monitored clinical trial. It may be challenging to administer all of the same measures in a less controlled study.

The study and the PMMSA also had several notable strengths. The tests of reliability, validity, and responsiveness followed expert and regulatory best practices using the intensive within-patient observations to account for the relatively small sample size. The use of the daily diary approach with short-recall period (24 h) is a notable difference from other PRO measures that have been used with mitochondrial disease patients and generic measures of symptoms. Perhaps because of this approach, the PMMSA fatigue score was more sensitive to treatment effects than other PRO measures, as described in a separate publication [25].

## Conclusion

The PMMSA is a content-valid PRO measure whose subdomain and individual item scores have been found to be reliable, construct-valid, and interpretable in patients with genetically confirmed mitochondrial disease, specifically, those with mitochondrial myopathy. Also, the PMMSA fatigue scores are suited for use as an independently scored subscale among this population. Other items can be used individually to comprehensively evaluate the patient’s symptom burden. The findings suggest that the PMMSA is a valuable tool to examine the patient’s perspective on those symptoms that negatively affect their QoL and activities of daily living in clinical trials.

## Supplementary Information

**Additional file 1. Supplemental Table 1:** Primary Mitochondrial Myopathy Symptom Assessment.

## Data Availability

The datasets generated and/or analyzed during the current study are proprietary to Stealth BioTherapeutics and are therefore not publicly available. Further details and results of the trial described in this study may be found in the following published article: Karaa A, Haas R, Goldstein A, Vockley J, Cohen BH. A randomized crossover trial of elamipretide in adults with primary mitochondrial myopathy. *J Cachexia Sarcopenia Muscle*. 2020 Aug;11(4):909–918.

## Abbreviations

2FS: Two-item fatigue scale (of the PMMSA)
3TUG: Triple Timed Up and Go
4FS: Fatigue 4 Scale (of the PMMSA)
6MWT: Six-minute walk test
CEFA: Categorical exploratory factor analysis
mtDNA: Mitochondrial DNA
nDNA: Nuclear DNA
Neuro-QoL: Quality of Life in Neurological Disorders
NMDAS: Newcastle Mitochondrial Disease Adult Scale
NMQ: Newcastle Mitochondrial Quality of Life measure
PhGA: Physician Global Assessment
PGA: Patient Global Assessment
PMD: Primary mitochondrial diseases
PMM: Primary mitochondrial myopathy
PMMSA: Primary Mitochondrial Myopathy Symptom Assessment
PRO: Patient-reported outcome
QoL: Quality of life
SARA: Scale for the Assessment and Rating of Ataxia
SD: Standard deviation
SEM: Standard error of measurement
TSS: Total symptom scale
VRS: Verbal rating scale

## References

[1] Parikh S, Karaa A, Goldstein A, Bertini ES, Chinnery PF, Christodoulou J (2019). Diagnosis of 'possible' mitochondrial disease: an existential crisis. J Med Genet.

[2] Gorman GS, Chinnery PF, DiMauro S, Hirano M, Koga Y, McFarland R (2016). Mitochondrial diseases. Nat Rev Dis Primers.

[3] DiMauro S (2013). Mitochondrial encephalomyopathies–fifty years on: the Robert Wartenberg Lecture. Neurology.

[4] DiMauro S, Schon EA (2003). Mitochondrial respiratory-chain diseases. N Engl J Med.

[5] DiMauro S (2006). Mitochondrial myopathies. Curr Opin Rheumatol.

[6] Mancuso M, McFarland R, Klopstock T, Hirano M (2017). International Workshop: Outcome measures and clinical trial readiness in primary mitochondrial myopathies in children and adults. Consensus recommendations. 16-18 November 2016, Rome, Italy. Neuromuscul Disord.

[7] Schaefer AM, McFarland R, Blakely EL, He L, Whittaker RG, Taylor RW (2008). Prevalence of mitochondrial DNA disease in adults. Ann Neurol Off J Am Neurol Assoc Child Neurol Soc.

[8] Chinnery PF, Pagon RA, Adam MP, Ardinger HH, Wallace SE, Amemiya A, Bean LJH (1993). Mitochondrial disorders overview. GeneReviews®.

[9] Gorman GS, Schaefer AM, Ng Y, Gomez N, Blakely EL, Alston CL (2015). Prevalence of nuclear and mitochondrial DNA mutations related to adult mitochondrial disease. Ann Neurol.

[10] Behin A, Salort-Campana E, Wahbi K, Richard P, Carlier RY, Carlier P (2015). Myofibrillar myopathies: State of the art, present and future challenges. Rev Neurol (Paris).

[11] Zolkipli-Cunningham Z, Xiao R, Stoddart A, McCormick EM, Holberts A, Burrill N (2018). Mitochondrial disease patient motivations and barriers to participate in clinical trials. PLoS ONE.

[12] Taylor RW, Turnbull DM (2005). Mitochondrial DNA mutations in human disease. Nat Rev Genet.

[13] Husted JA, Gladman DD, Farewell VT, Cook RJ (2001). Health-related quality of life of patients with psoriatic arthritis: a comparison with patients with rheumatoid arthritis. Arthritis Care Res.

[14] Orsucci D, Calsolaro V, Siciliano G, Mancuso M (2012). Quality of life in adult patients with mitochondrial myopathy. Neuroepidemiology.

[15] United Mitochondrial Disease Foundation. Voice of the Patient Report: Mitochondrial Disease: Adults with Myopathy, Children with Neurologic Symptoms. United Mitochondrial Disease Foundation; 2018. p. 1–78.

[16] Goldstein A, Rahman S (2020). Seeking impact: Global perspectives on outcome measure selection for translational and clinical research for primary mitochondrial disorders. J Inherit Metabol Dis.

[17] Pfeffer G, Horvath R, Klopstock T, Mootha VK, Suomalainen A, Koene S (2013). New treatments for mitochondrial disease-no time to drop our standards. Nat Rev Neurol.

[18] National Institutes of Health (2017). ClincalTrials.gov. Accessed 9/19/2017.

[19] Schaefer AM, Phoenix C, Elson JL, McFarland R, Chinnery PF, Turnbull DM (2006). Mitochondrial disease in adults: a scale to monitor progression and treatment. Neurology.

[20] Elson JL, Cadogan M, Apabhai S, Whittaker RG, Phillips A, Trennell MI (2013). Initial development and validation of a mitochondrial disease quality of life scale. Neuromuscul Disord.

[21] Patrick DL, Burke LB, Gwaltney CJ, Leidy NK, Martin ML, Molsen E (2011). Content validity-establishing and reporting the evidence in newly developed patient-reported outcomes (PRO) instruments for medical product evaluation: Ispor PRO good research practices task force report: part 2-assessing respondent understanding. Value Health.

[22] Patrick DL, Burke LB, Gwaltney CJ, Leidy NK, Martin ML, Molsen E (2011). Content validity-establishing and reporting the evidence in newly developed patient-reported outcomes (PRO) instruments for medical product evaluation: ISPOR PRO good research practices task force report: part 1-eliciting concepts for a new PRO instrument. Value Health.

[23] US Department of Health and Human Services, Food and Drug Administration, Center for Drug Evaluation and Research, Center for Biologics Evaluation and Research, & Center for Devices and Radiological Health (2009). Guidance for Industry Patient-Reported Outcome Measures: Use in Medical Product Development to Support Labeling Claims. Silver Spring, MD: Office of Communications, Division of Drug Information.

[24] Gwaltney C, Stokes J, Aiudi A, Mazar I, Ollis S, Love E (2020). Development of a patient-reported outcome questionnaire to evaluate primary mitochondrial myopathy symptoms: the primary mitochondrial myopathy symptom assessment. J Clin Neuromuscul Dis.

[25] Karaa A, Haas R, Goldstein A, Vockley J, Cohen BH (2020). A randomized crossover trial of elamipretide in adults with primary mitochondrial myopathy. J Cachexia Sarcopenia Muscle.

[26] Karaa A, Haas R, Goldstein A, Vockley J, Weaver WD, Cohen BH (2018). Randomized dose-escalation trial of elamipretide in adults with primary mitochondrial myopathy. Neurology.

[27] Cella D, Lai JS, Nowinski CJ, Victorson D, Peterman A, Miller D (2012). Neuro-QOL: brief measures of health-related quality of life for clinical research in neurology. Neurology.

[28] Enright PL (2003). The six-minute walk test. Respir Care.

[29] Podsiadlo D, Richardson S (1991). The timed "Up & Go": a test of basic functional mobility for frail elderly persons. J Am Geriatr Soc.

[30] Schmitz-Hubsch T, du Montcel ST, Baliko L, Berciano J, Boesch S, Depondt C (2006). Scale for the assessment and rating of ataxia: development of a new clinical scale. Neurology.

[31] Wirth RJ, Edwards MC (2007). Item factor analysis: current approaches and future directions. Psychol Methods.

[32] Shields A, Coon C, Hao Y, Krohe M, Yaworsky A, Mazar I (2015). Patient-reported outcomes for US oncology labeling: review and discussion of score interpretation and analysis methods. Expert Rev Pharmacoeconomics Outcomes Res.

[33] Wyrwich KW, Norquist JM, Lenderking WR, Acaster S, Industry Advisory Committee of International Society for Quality of Life Research (ISOQOL) (2013). Methods for interpreting change over time in patient-reported outcome measures. Qual Life Res.

[34] Cappelleri JC, Zou KH, Bushmakin AG, Alvir JMJ, Alemayehu D, Symonds T (2013). Patient-reported outcomes: measurement, implementation and interpretation.

[35] Brown TA (2015). Confirmatory factor analysis for applied research.

[36] Cohen J (1988). Statistical power analysis for the behavioral sciences.

[37] Cohen J (1992). A power primer. Psychol Bull.

[38] Peipert JD, Hays RD, Cella D (2022). Likely change indexes improve estimates of individual change on patient-reported outcomes. Qual Life Res.

[39] Revicki D, Hays RD, Cella D, Sloan J (2008). Recommended methods for determining responsiveness and minimally important differences for patient-reported outcomes. J Clin Epidemiol.

[40] Benjamin K, Vernon MK, Patrick DL, Perfetto E, Nestler-Parr S, Burke L (2017). Patient-reported outcome and observer-reported outcome assessment in rare disease clinical trials: an ISPOR COA emerging good practices task force report. Value Health.

[41] Wild D, Grove A, Martin M, Eremenco S, McElroy S, Verjee-Lorenz A (2005). Principles of good practice for the translation and cultural adaptation process for patient-reported outcomes (pro) measures: report of the ISPOR task force for translation and cultural adaptation. Value Health.

