# Supplementary material for: Psychometric performance of the Primary Mitochondrial Myopathy Symptom Assessment (PMMSA) in a randomized, double-blind, placebo-controlled crossover study in subjects with mitochondrial disease
Source: J Patient Rep Outcomes. 2022 Dec 23;6:129. doi: 10.1186/s41687-022-00534-y (PMC9789285; doi:10.1186/s41687-022-00534-y)
Supplement: Supplementary file 1 — Additional file 1. Supplemental Table 1: Primary Mitochondrial Myopathy Symptom Assessment. [file 41687_2022_534_MOESM1_ESM.docx]

Supplemental Table 1: Primary Mitochondrial Myopathy Symptom Assessment

|  | Not at all | Mild | Moderate | Severe |
| --- | --- | --- | --- | --- |
| During the past 24 hours, how severe was your worst feeling of tiredness at rest? | □ | □ | □ | □ |
| During the past 24 hours, how severe was your worst feeling of tiredness during activities? | □ | □ | □ | □ |
| During the past 24 hours, how severe was your worst feeling of muscle weakness at rest? | □ | □ | □ | □ |
| During the past 24 hours, how severe was your worst feeling of muscle weakness during activities? | □ | □ | □ | □ |
| During the past 24 hours, how severe were your worst balance problems? | □ | □ | □ | □ |
| During the past 24 hours, how severe were your worst vision problems? | □ | □ | □ | □ |
| During the past 24 hours, how severe was your worst abdominal discomfort (feeling nauseous, bloated, or in pain)? | □ | □ | □ | □ |
| During the past 24 hours, how severe was your worst muscle pain? | □ | □ | □ | □ |
| During the past 24 hours, how severe was your worst numbness? | □ | □ | □ | □ |
| During the past 24 hours, how severe was your worst headache? | □ | □ | □ | □ |
